# Growth Failure and Primary Amenorrhea: Endocrine Dysfunction as a Late Presentation of Crohn's Disease

**DOI:** 10.7759/cureus.42020

**Published:** 2023-07-17

**Authors:** Suganthika D Muthusamy, Navaneethakrishnan Suganthan, Vathulan Sujanitha

**Affiliations:** 1 Internal Medicine, Postgraduate Institute of Medicine, Colombo, LKA; 2 Medicine, University of Jaffna, Jaffna, LKA

**Keywords:** endocrine manifestations, inflammatory bowel disease, primary amenorrhea, growth failure, crohn’s disease

## Abstract

Crohn’s disease (CD) is a chronic inflammatory bowel disease involving entire gastrointestinal tract, most commonly affecting terminal ileum and colon. It usually presents with gastrointestinal symptoms like bloody diarrhea, fever and loss of weight. The clinical course of CD includes gastrointestinal complications like fistulas, abscesses and perianal disease.

Inflammatory bowel diseases (IBD) are usually diagnosed during childhood and adolescence, majority during puberty and pubertal growth spurt. Various extraintestinal manifestations may be a presentation of CD that poses a diagnostic challenge. Growth failure is an important complication of IBD rather than a manifestation. Herein we present a case of a 16-year-old Sri Lankan girl presenting with growth failure and primary amenorrhea. She had minimal gastrointestinal symptoms. She also had microcytic anemia with marginally elevated inflammatory markers and hormonal profile. She underwent colonoscopy and was diagnosed to have Crohn’s disease confirmed by ileal biopsy. On initiation of treatment with immunosuppressants, she attained menarche, although no improvement in height was observed.

## Introduction

Crohn’s disease (CD) and ulcerative colitis are two major inflammatory bowel diseases (IBD), more prevalent in developed countries [1]. The prevalence of IBD in Sri Lanka is lower compared to the western population. The annual incidence of ulcerative colitis and CD in Sri Lanka is 1.0 per 100,000 and 0.6 per 100,000 population, respectively [2]. The pathogenesis is uncertain although interactions between genetic, environmental, gut microbial factors are postulated [3]. Symptoms depend on the location and the severity of the disease. Patients may have flare-ups of the disease requiring aggressive treatment. Extraintestinal manifestations occur in about one-third of patients with IBD and may precede the onset of gastrointestinal symptoms by many years [4]. Considering the paediatric population, the mean delay in diagnosis of CD is 4-24 months, while its 2-18 months for ulcerative colitis. When presenting with extraintestinal features, a high level of suspicion is needed to diagnose CD. Here, we report the case of a young school girl presenting with features of growth failure and primary amenorrhea as an atypical presentation of CD.

## Case presentation

A 16-year-old girl presented with loss of appetite and weight loss of four-year duration. Due to the COVID-19 pandemic as well as her poor socioeconomic background, the family did not seek medical treatment for the said symptoms. She also complained of being short in class and not having attained menarche. She did not have fever, joint pains, oral ulcers, hair loss, red eyes, back pain or symptoms suggestive of hyperthyroidism or hypothyroidism. She had a history of occasional loose stools for the past six months, which were small in quantity, watery, and not associated with blood, mucus or abdominal pain. Loose stools settled spontaneously without any treatment. Four years ago, she was evaluated by the pediatric team for weight loss and anemia and was treated with hematinics, as for iron deficiency nutritional anemia, but lost follow-up to treatment in four months. She was not diagnosed with any congenital anomalies during antenatal or postnatal periods and had a normal, healthy childhood until she presented with the above-mentioned symptoms. She had age-appropriate milestone achievements and denied recurrent childhood infections. Her mother’s age of puberty was 13 years. Her school performance was also found to be good. Her two younger siblings had age-appropriate heights, and her mother’s and father’s heights were 147 cm and 157 cm, respectively. On physical examination, her weight was recorded as 30 kg, height 143 cm and BMI 14.3 kg/m²; blood pressure was 110/70 mmHg. Apart from mild bilateral pitting pedal edema, her general examination was unremarkable. The abdomen was soft, non-tender with no organomegaly or lymphadenopathy, and respiratory, cardiovascular and thyroid examinations were normal. No breast buds or secondary sexual hair was noted. Laboratory findings are given in Table 1.

**Table 1:** Laboratory findings with reference values AST: aspartate aminotransferase; ALT: alanine aminotransferase; ALP: alkaline phosphatase; free T4: free thyroxine

Laboratory investigation	Results (reference ranges)
Hemoglobin	10.3 g/dL (12–15 g/dL)
Mean corpuscular volume	68 fL (80–100 fL)
Mean corpuscular hemoglobin	21 pg (27–34 pg)
White blood cell count	5.5 x 10^9^/L (4–10 x 10^9^/L)
Platelet count	535 x 10^9^/L (150–400 x 10^9^/L)
Erythrocyte sedimentation rate	30 mm/hour, 1sthour (<10 mm/hour, 1sthour)
C-reactive protein	10 mg/dL (<6 mg/dL)
Total protein	61 g/L (64–82 g/L)
Albumin	21 g/L (34–50 g/L)
AST	22 U/L (15–37 U/L)
ALT	43 U/L (16–63 U/L)
ALP	176 U/L (30–180 U/L)
Corrected serum calcium	2.4 mmol/L (2.1–2.5 mmol/L)
Magnesium	0.8 mmol/L (0.7–1.0 mmol/L)
Ferritin	21.4 mg/L (24–336 mg/L)
Thyroid stimulating hormone	1.26 mIU/L (0.4–4.6 mIU/L)
Free T4	1.17 ng/dL (0.7–2.19 ng/dL)
Cortisol	333 nmol/L (123–626 nmol/L)
Insulin-like growth factor 1	135 ng/mL (106–379 ng/mL)
Estradiol	341 pmol/L (71–1145 pmol/L)
Prolactin	272 mIU/L (64–395 mIU/L)
Luteinizing hormone	2.58 mIU/L (2.58–12.1 mIU/L)
Follicle stimulating hormone	9.63 mIU/L (1.98–11.6 mIU/L)

**Figure 1:**
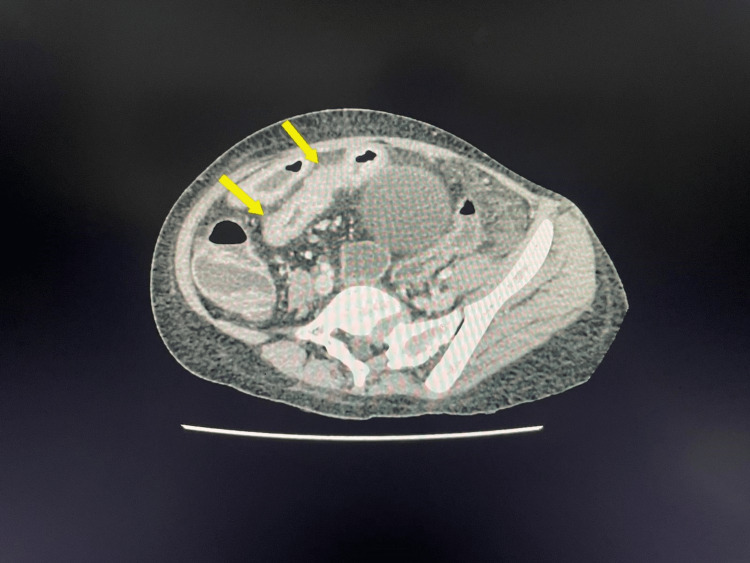
Contrast enhanced computed tomography (CECT) of the abdomen and pelvis The yellow arrows show long and short segments of small bowel wall thickening with abnormal mucosal enhancement (skip lesions) mainly involving the ileal loops.

An ultrasound scan of the abdomen showed no organomegaly or lymphadenopathy but showed small uterus and ovaries for the corresponding age. However, contrast enhanced computed tomography (CECT) of the abdomen and pelvis showed long and short segments of small bowel wall thickening with abnormal mucosal enhancement (skip lesions) mainly involving the ileal loops suggestive of tuberculous enteritis or Crohn’s disease (Figure 1).

**Figure 2:**
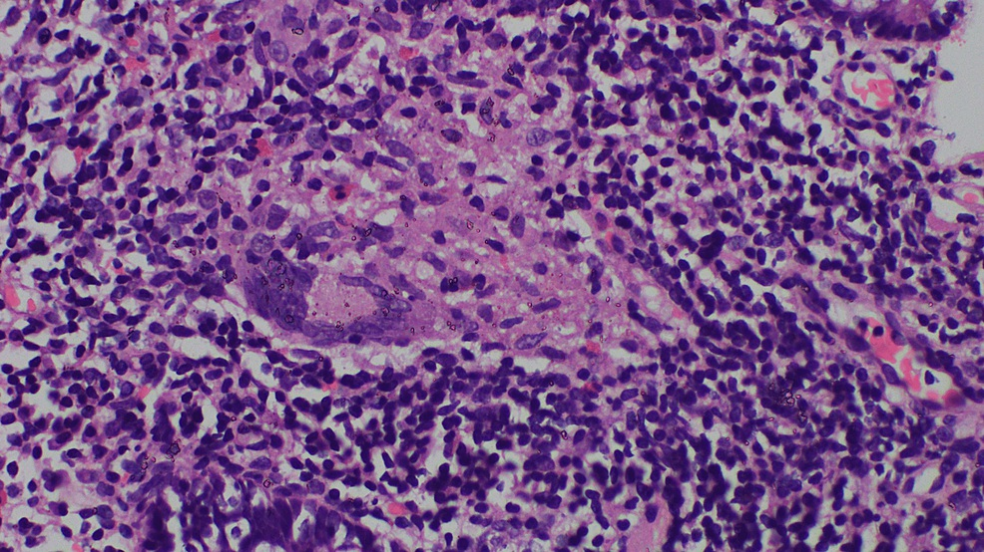
Moderate chronic active colitis with ulcerations and granuloma formation

Upper gastro-endoscopy was normal up to second segment of the duodenum. Colonoscopy showed ileal ulcer, stricture with friable mucosa with contact bleeding and edematous microvilli. Biopsy of the terminal ileum and of ileo-caecal valve showed moderate active colitis with ulceration and granuloma formation (Figure 2) and focal active colitis in the right and left colon. The Mantoux test was negative; transglutaminase antibody test results were also negative. X-ray radiographs of hands showed normal bone maturation for age. Considering the clinical picture, imaging and histology, a diagnosis of Crohn’s disease manifesting as growth failure due to associated nutritional deficiency was made. She was treated with intravenous and oral steroids. On follow-up at six months of treatment, she had attained menarche, although there was no major improvement in linear growth.

## Discussion

Inflammatory bowel disease is an autoimmune inflammatory disease primarily involving the gastrointestinal tract [5]. There are three subtypes, namely, ulcerative colitis, Crohn’s disease and IBD unclassified [6]. Small bowel inflammation in CD exhibits an increased level of proinflammatory cytokines such as interferon-gamma (IFN-γ) and interleukin (IL) 17A (produced by Th1 and Th17 cells, respectively) [7]. Common extraintestinal manifestations include arthropathies, erythema nodosum, pyoderma gangrenosum, primary biliary cholangitis, uveitis and episcleritis. Other organ involvement is relatively rare. As a child, our patient was managed for iron deficiency anemia with hematinics suspecting a nutritional deficiency. Anemia is part of many diseases. Concluding anemia as a nutritional deficiency only, in a young girl with a relatively good nutrition intake, should be a diagnosis of exclusion. She was lost to follow-up and sought medical attention only when her growth and puberty were not attained as expected for her age. Her initial symptom analysis was inconclusive. Only on repeated questioning, she came up with the history of occasional loose stools that pointed towards a possible gastrointestinal pathology. An upper and lower gastrointestinal endoscopy was performed suspecting celiac disease. Her colonoscopy showed thickened bowel loops with skip lesions and the biopsy showed granuloma formation with active colitis, which is not a typical feature of celiac disease. In Sri Lanka, tuberculosis (TB) is a very important disease to rule out with this histological finding, and TB screening came out negative. Further testing with calprotectin could not be done due to financial constraints.

Growth failure is a common extraintestinal manifestation of Crohn’s disease with a multifactorial etiology. In active CD, insulin-like growth factor 1 (IGF-1) levels are low with normal circulating growth hormone (GH) levels indicating GH resistance as a mechanism [8]. Furthermore, inflammatory markers like IL-1 and tumor necrosis factor (TNF), and nutritional deficiency also play an important role [8]. The lack of multiple nutrients may be due to constitutional symptoms of loss of appetite, malabsorption or intestinal bleeding and dietary restrictions [9]. Iron deficiency anemia is a very common finding in these patients. They have associated zinc, vitamin K and vitamin D deficiencies that can further contribute to growth failure [10]. In our patient, when comparing her height to her mother’s and father’s heights, the short stature could be considered as familial if all the above-mentioned factors are corrected and still no improvement is seen in growth. Delayed puberty is frequently found in adolescents with chronic inflammatory disease and it is particularly common in females with CD [11]. The cytokines also inhibit sex steroid production with direct action on glands [11]. There is a suppression of gonadotropin-releasing hormone (GnRH) secretion with resultant reduction in luteinizing hormone (LH) and follicle stimulating hormone (FSH) [12]. On initiation of treatment with steroids, the patient attained puberty that specifies the autoimmunity behind. In this case, the patient had a loss of appetite and a six-month history of occasional loose stools along with malabsorption due to active colitis that could have contributed to macro- and micro-nutrient deficiencies.

## Conclusions

Inflammatory bowel diseases are inflammatory diseases with varying presentations. Although gastrointestinal manifestations are the common presenting symptoms, we should remain highly vigilant for extraintestinal manifestations as well. Rarely, they may present with a complication of the disease rather than the disease itself, like in our case. Good history taking is needed to bring out subtle symptoms that the patient might regard as normal. Another important consideration when it comes to pubertal delay in adolescent children is that it affects bone mineralization and has psychological impacts, when compared with peers.
